# Vine-Canes as a Source of Value-Added Compounds for Cosmetic Formulations

**DOI:** 10.3390/molecules25132969

**Published:** 2020-06-28

**Authors:** Manuela M. Moreira, Francisca Rodrigues, Olena Dorosh, Diana Pinto, Paulo C. Costa, Jaroslava Švarc-Gajić, Cristina Delerue-Matos

**Affiliations:** 1 REQUIMTE/LAQV, Instituto Superior de Engenharia do Instituto Politécnico do Porto, Rua Dr. António Bernardino de Almeida, 431, 4249-015 Porto, Portugal; francisca.rodrigues@graq.isep.ipp.pt (F.R.); olena.dorosh@graq.isep.ipp.pt (O.D.); diana.pinto@graq.isep.ipp.pt (D.P.); cmm@isep.ipp.pt (C.D.-M.); 2 REQUIMTE/UCIBIO, MedTech-Laboratory of Pharmaceutical Technology, Department of Drug Sciences, Faculty of Pharmacy, University of Porto, Rua de Jorge Viterbo Ferreira nº. 228, 4050-313 Porto, Portugal; pccosta@ff.up.pt; 3 Faculty of Technology, University of Novi Sad, Bulevar cara Lazara 1, 21000 Novi Sad, Serbia; jsgajic@gmail.com

**Keywords:** vine-canes, subcritical-water extraction, antioxidants, cytotoxicity, cosmetic products

## Abstract

The majority of works about vine-canes are focused on the evaluation of their chemical composition and antioxidant potential. To the best of our knowledge, the possible applications of produced extracts in cosmetic formulations have never been explored. The aim of the present study was to evaluate the antioxidant properties of vine-canes subcritical water extracts for use as active ingredients in the cosmetic industry. For that, the phenolic content and antioxidant activity of six vine-cane varieties, namely Alvarinho and Loureiro from the Minho region and Touriga Nacional and Tinta Roriz (TR) from both the Douro and Dão regions, were evaluated through spectrophotometric and chromatographic methods. All extracts presented similar antioxidant activity and the highest phenolic content was reported for TR variety from the Douro region (33.7 ± 1.9 mg GAE/g dw). The capacity of vine-cane extracts to capture reactive oxygen species superoxide (O_2_^∙−^) was also studied, with the highest IC_50_ value being obtained for Loureiro variety (56.68 ± 2.60 µg/mL). Furthermore, no adverse effects on HaCaT and HFF-1 dermal cell lines in concentrations below 100 and 1000 μg/mL, respectively, were determined. Finally, Loureiro vine-cane extract was incorporated into a topical formulation, and physical and microbiological properties were within expected values, demonstrating that vine-canes extracts can be successfully incorporated in cosmetic products.

## 1. Introduction

The wine making industry is a growing sector around the world, which produces substantial amounts of wastes, including vine-canes. In Portugal, after the harvest season, around 1.2 to 3.5 t/ha of vineyard pruning residues are produced annually [1,2], which are usually incinerated or incorporated back into the field as organic fertilizer, representing an economical and environmental issue for the wine industry.

Recently, several studies have reported that vine-canes can be used as potential sources of antioxidant compounds [2,3,4,5], and their valorization is attracting a lot of interest. Indeed, it is widely described in the literature, that besides phenolic compounds’ antioxidant properties, they are also capable to directly inhibit enzymes responsible for the skin ageing process, namely tyrosinase, collagenase (MMP-1) and elastase [6,7]. As far as we know, several studies have already exploited the use of grape or wine extracts as cosmetic ingredients due to their renowned antioxidant activities [7]. However, the incorporation of vine-cane extracts in cosmetic products was never explored. Therefore, the recovery of bioactive compounds, such as polyphenols, from vineyard pruning wastes could represent a potential application for their re-use contributing to the sustainable development of the wine industry.

Recently, obtaining bioactive compounds from agro-forestry wastes with environmentally friendly extraction techniques is a hot topic in the food and pharmaceutical industries. In fact, the choice of an appropriate extraction technique is of huge importance as it influences the type and amount of bioactive compounds extracted, as well as their bioactivity [8]. Alternative extraction techniques have been used for the recovery of phenolic compounds from vineyard pruning residues, including hydrothermal treatment at high temperatures [1,9], ultrasound-assisted extraction [2], alkaline hydrolysis treatments [10], microwave-assisted extraction (MAE) [3,4,5] and subcritical water extraction (SWE) [3,11]. Among these alternatives, SWE is one of the most promising innovative extraction techniques that has been developed and employed for the extraction of highly active compounds from natural products [3,12,13,14]. This green extraction technique has captured more and more attention due to its safety, efficiency, and low environmental impact. Additionally, SWE can cause a modification effect on the molecular structure, which can improve biological properties of bioactive compounds recovered demonstrating its potential value for application in cosmetic industry [11,15].

The main goal of the present work was to investigate the possibility to incorporate vine-cane extracts in cosmetic products. For that, the antioxidant properties from six different vine-cane varieties, namely Touriga Nacional (TN) and Tinta Roriz (TR) from the Dão and Douro regions and Alvarinho and Loureiro from Minho region, were determined and compared. The recovery of bioactive compounds was performed employing SWE that enable the production of safe extracts for incorporation into a topical formulation. For this purpose, phytochemical and antioxidant activity characterization, as well as the cytotoxicity in different kinds of skin cell types, namely keratinocytes and fibroblasts, were performed for all the obtained extracts. Then, after selecting the most promising vine-cane extract, a topical formulation was prepared and its physical characteristics, namely pH, color, texture and rheological behavior, as well as microbiological properties were evaluated. To the best of our knowledge, there is no published data on the cytotoxic potential of vine-cane subcritical water extracts and their incorporation in a topical formulation. Therefore, the evaluation of these properties in different vine-canes varieties remains an important challenge in understanding the possible benefits of their application in cosmetic products.

## 2. Results and Discussion

### 2.1. Phytochemical and Antioxidant Activity Characterization

#### 2.1.1. Total Phenolic and Flavonoid Content

The total phenolic and flavonoid content of the six vine-cane subcritical water extracts are summarized at Table 1.

Regarding the phenolic content, the obtained values ranged from 27.38 ± 1.40 to 32.49 ± 2.70 mg GAE/g dw, and statistically significant differences were observed between the six vine-cane varieties studied (*p* < 0.05). As far as we know, several studies were carried out in the last few years on the valorization of vine-canes [2,3,4,5,16], although, none of these studies report the incorporation of the obtained extracts into a topical formulation. Furthermore, most of the reported studies were conducted with different extraction techniques and employing organic solvents. Only Gabaston and co-workers [11] reported the use of SWE for the recovery of complex stilbenes from three vine by-products, namely canes, wood and roots. Concerning the vine-canes, the authors reported that the highest total stilbene yield was obtained at 160 °C and 5 min extraction time (3.62 g/kg dw). In the present study, phenolic compounds belonging to different families were also identified and quantified by HPLC (Table 2), although, a direct comparison with the results from the previous authors cannot be made as we only quantified the stilbene resveratrol.

Despite from abovementioned facts, in order to validate the potential of the produced subcritical water extracts to be incorporated in cosmetic formulations, a comparison of the TPC and TFC obtained by other authors with different extraction techniques was performed. For example, Jesus et al. [4] recently optimized the extraction of polyphenol compounds from vine pruning residues of the Loureiro variety by conventional heating (21.7 mg GAE/g dw) and microwave-assisted treatments (23.7 mg GAE/g dw), and reported lower values than the ones obtained in this study for the same vine-cane variety (29.26 ± 1.47 mg GAE/g dw). These differences may be justified by the distinct extraction techniques and conditions employed (conventional heating: 80 °C, 120 min and 45% of ethanol; microwave-assisted process: 120 °C, 5 min and 60% of ethanol). Furthermore, the higher efficiency of the SWE may be explained by the fact that part of the phenolic compounds are entrapped within the lignocellulosic structure, and their release requires hydrolysis of the lignocellulosic structure, which can occur by the application of SWE contributing to the phenolics content recorded [9,17]. In our previous study [3], a comparison between conventional extraction and SWE was performed and higher TPC values were also obtained for the subcritical extracts. In fact, SWE was employed to TN and TR vine-canes varieties from the Dão region collected in November 2015, and the TPC values obtained were 22.5 ± 1.8 and 31.9 ± 1.6 mg GAE/g dw for TN and TR varieties, respectively. Comparing the vine-canes varieties from the same region, the main difference between the two studies was the samples pruning period which may be the main responsible for the differences in the TPC values. According to Matos et al. [7] these differences between varieties from distinct years can be explained by the diverse composition of the raw material used, since crops from different years may present considerable variability in sugar content and nutritional composition. Further, environmental and microclimatic conditions, plant disease or soil type can also influence the phenolics concentration [18]. In another study, Gullón et al. [9] reported the TPC of vine-canes extracts from *V. vinifera* Hondarribi Zuri variety (Spain) obtained from the refining of autohydrolysis liquors, which is an extraction technique similar to the one used in the present study. The authors mixed the vine-canes with water at a liquid-solid ratio of 8 g/g dw and heated in a reactor with a temperature profile between 180 and 215 °C. After cooling, the liquor was extracted with ethyl acetate at a liquor:solvent ratio of 1:3 (*v*/*v*) at room temperature under stirring for 15 min, and the TPC values ranged from 8.8 to 22.5 mg GAE/g dw [9]. Despite from the increase reported by these authors in the yields of phenolics (from 44% at 180 °C to 80% at 210 °C), the results obtained in the present study were higher (Table 1). As previously mentioned, the differences in the TPC values between the reported studies and ours could be explained by the different extraction techniques and conditions employed, as well as by the vine-cane varieties studied [4].

Flavonoids represent one of the biggest classes of plant polyphenols, and usually their content its correlated with the TPC. In the present study a correlation coefficient of 0.93 between the two assays were obtained (data not shown), demonstrating the good fit for the spectrophotometric assays performed. According to Table 1, Alvarinho variety presented the lowest result (6.03 ± 0.34 mg EE/g dw), and on the contrary TR variety from Douro region presented the highest flavonoid content (10.07 ± 0.94 mg EE/g dw). As previously observed for the TPC assay, these results suggest that the vine-cane variety seems to exert a significant effect on the extraction efficiency, with the vine-cane varieties belonging to Minho region presenting the lowest flavonoid content. Comparing the TFC of the present study with our previous reported values [3], it was observed approximately a 2-fold lower flavonoid content, which may be related to the sample data collection as it was from different years. Further, different environmental and microclimatic conditions or a plant disease could be the main responsible for the differences observed.

#### 2.1.2. Identification and Quantification of Individual Phenolic Compounds by HPLC-DAD

An HPLC analysis was performed to identify and quantify the main phenolic compounds present in the six vine-cane varieties (Table 2). The retention time and UV–vis spectra of detected peaks were compared with those obtained for their pure standards, and the quantification was made at 280, 320 and 360 nm depending on the maximum absorption from the phenolic compound.

In all cases, the extracts obtained from the six varieties had the same qualitative composition, differing only in quantitative composition, depending on the vine-cane studied. It was observed that TR variety from the Douro region presents the highest amount of individual phenolic compounds quantified, while Alvarinho from the Minho region presented the lowest (1610 vs. 1135 mg/100 g dw, respectively). In general, the phenolic profiles of vine-canes were dominated by three polyphenols classes, namely phenolic acids, flavonoids and stilbenes. It was observed that phenolic acids were the major contributors to the total amount of all quantified compounds, corresponding at least to 63%. Gallic acid exerts the major contribution, with levels ranging from 527 ± 26 to 1014 ± 51 mg/100 g dw for TN Dão and TR Douro, respectively. On the contrary, 4-hydroxybenzoic, *p*-coumaric and cinnamic acids were present in lowest amount in all varieties studied, with values below 6.41 mg/100 g dw. In the case of compounds belonging to the flavonoid family, their contribution ranged from 23 (TR from the Douro region) to 36% (TN from the Dão region), with (+)-catechin and myricetin representing more than half of this value. In fact, the amount of individual flavonoids compounds quantified in the Loureiro variety could be the main responsible for their antioxidant properties (Table 1). It must be highlighted the content of (+)-catechin, which radical scavenging properties are well described in literature, that was the highest for the Loureiro variety (289 ± 14 mg/100 g dw). In fact, these data could help us to understand why the Loureiro variety presented the lowest total phenolic and flavonoid content but the highest capacity to scavenge superoxide anion (Table 1). Additionally, it must be highlighted that phenolic compounds present in vine-cane extracts may exert their antioxidant capacity individually as well as synergistically.

These phenolic compounds, occurring in vine-canes, have been well identified in previous studies [2,3,4,5]. Although, different amounts have been quantified depending on the variety studied, growing conditions (altitude, climate, soil, and agriculture practices) as well as the extraction conditions employed. To our best knowledge, just one study reported the use of SWE for the recovery of bioactive compounds from vine-canes and it was focused on stilbenes extraction [11]. Only in our previous study [3], SWE was employed to TN and TR varieties from the Dão region collected in 2015, and gallic acid, (+)-catechin and myricetin were also identified as the major contributors for the phenolic content of obtained extracts; however TN variety presented the highest amount which is not in agreement with the present study, demonstrating that the pruning season can exert an influence in the individual quantified polyphenols. Jesus et al. [4] also performed an HPLC analysis of the Loureiro extracts obtained by MAE and CE, and despite having reported a phenolic profile very similar to the one of the present study, the levels obtained were very different. These authors reported that apigenin was the most abundant flavonoid (207.9 mg/100 g dw), while naringenin (6.2 mg/100 g dw), ferulic acid (6.9 mg/100 g dw) and epicatechin + *p*-coumaric acid (6.7 mg/100 g dw) were present in lower amounts. In another study, Gullón et al. [9] employed a similar extraction procedure to the one of the present work (autohydrolysis treatment followed by a ethyl acetate solid-liquid extraction) reporting that the major phenolic compounds identified were gallic acid, 5-hydroxymethylfurfural (5-HMF), synaptic acid, furfural, catechol, 4-hydroxybenzoic acid, vanillic acid and syringic acid. Sánchez-Gómez et al. [5] also tested different extraction techniques, namely conventional solid-liquid extraction, solid-liquid dynamic extraction, and MAE, employing water as solvent and also reported that the flavonoids (+)-catechin and (−)-epicatechin, followed by ellagic and gallic acids, were the most abundant phenolics in all the obtained vine-cane extracts.

#### 2.1.3. Antioxidant Activity Characterization

Radical scavenging activity is an important parameter due to the effects that free radicals exerts on biological systems, including human tissues. In the present study, the antioxidant properties of vine-cane extracts were evaluated by DPPH and reducing power assay, while the radical scavenging activity against oxygen species was screened by superoxide anion and peroxyl radicals scavenging assays.

#### 2.1.4. FRAP and DPPH Assays

Considering the antioxidant activity, different methodologies are available being advisable the use of at least two. In the present study, the DPPH scavenging activity and the ferric-reducing antioxidant assays were used to evaluate the antioxidant activity (Table 1).

The reducing capacity of extracts, determined by FRAP assay, can serve as an indicator of their potential antioxidant activity. In Table 1 are showed the FRAP values obtained for the vine-cane extracts from six different varieties. The highest antioxidant activity was observed for TR variety from the Douro region, which was not statistically different from the Dão region (*p* > 0.05). On the other hand, the lowest FRAP activity was recorded for TN variety from the Dão region (29.99 ± 1.83 mg AAE/g dw), being statistical different from the other vine-cane varieties evaluated (*p* < 0.05).

Regarding the ability of the vine-cane extracts to scavenge the DPPH radicals it was significantly higher for vine-cane varieties from the Minho region (*p* < 0.05), as shown in Table 1. These results were not in agreement with the results observed in the previous employed assays in the characterization of vine-cane extracts. DPPH values obtained for vine-cane varieties from the Minho region were at least 1.6-fold higher than the ones determined for the other studied varieties. In fact, the statistical analysis showed differences between the six vine-canes (*p* < 0.05), indicating different behaviors by the evaluated extracts. As can be seen through the observation of Table 1, it can be established three different groups within the six varieties studied separating them by the region of collection, with the Dão region presenting the lowest DPPH values, followed by the Douro and Minho regions with the highest capacity to scavenge DPPH free radicals. Usually, extracts that contain a high amount of polyphenols also exhibit high antioxidant activity [16]; although, in this case no correlation was observed between TPC or TFC and DPPH assay (data not shown). Additionally, no correlation was also obtained for DPPH and FRAP assays. In this case, despite from both assays being described as electron transfer (ET)-based assays, some authors have also reported that DPPH assay can be considered as a mixed hydrogen atom transfer (HAT)/ET-based assay [19]. This difference between the assays principles could be a possible explanation for the reported variations in the present work for the vine-cane varieties studied, as the compounds present in the obtained subcritical water extracts could present different behavior for diverse spectrophotometric assays employed. In these cases, the results discussion must be done with precaution, as the extract effects depends on the combinatory and synergic actions of all their constituents. Therefore, one possible explanation could be the presence of some compounds which were not quantified as polyphenolic compounds in the TPC assay that could contribute to the observed effects. One possibility can be isoflavones compounds, which were not identified and quantified by HPLC analyses, and can further increase the chances of using this extract in skin care products [6].

Comparing the obtained FRAP and DPPH values from the present study with the ones from our previous report [3] it was observed, for the same vine-cane variety from the Dão region, an increase at least of 1.5-fold in FRAP values and 4-fold lower values in the case of DPPH assay. Although, similar capacities to scavenge the DPPH radicals to the ones of the present study were reported by Gullón et al. [9], with the highest values being obtained for the ethyl acetate extracts from the vine-cane autohydrolysis liquors at 215 °C (10.5 mg TE/g dw). Jesus and co-workers [4] also determined the antioxidant activity of vine pruning extracts from the Loureiro variety obtained by different extraction techniques (conventional and microwave-assisted extractions) reporting a superior capacity to reduce the DPPH radicals for conventional extraction (24 mg TE/g dw) and for microwave-assisted extraction (43 mg TE/g dw). However, we must keep in mind that a direct comparison of the antioxidant activities of wine derivatives with the literature is difficult due to the grape variety discrepancy and the multiplicity of extractive techniques and quantification methods employed [4].

#### 2.1.5. Reactive Oxygen Species Scavenging Assays

Unlike DPPH radicals, which are less relevant to the biological systems, ROS are one of the driving causes of skin aging [20,21,22]. Therefore, assessing the antioxidant activity of samples towards different biologically relevant radical species, namely superoxide (O_2_^∙−^) and peroxyl (ROO^∙^) radicals, is determinant for cosmetic applications.

Table 1 also summarizes the in vitro effect of vine-cane subcritical water extracts on the scavenging of ROS. In what concerns to O_2_^∙−^, Loureiro variety showed the highest scavenging efficiency (IC_50_ = 56.67 ± 2.59 µg/mL), while for TN from the Dão region was obtained the lowest O_2_^∙−^ reducing capacity. The most promising results obtained for the Loureiro variety may be explained by the amount of individual phenolic compounds, namely gallic acid and catechin, quantified by HPLC/DAD in comparison to the levels of the other vine-cane varieties tested (Table 2). Despite the best capacity of the Loureiro extracts for scavenging superoxide radical it presented a lower ability than any of the positive controls tested, revealing that a higher extract concentration is needed to exhibit the same scavenging capacity than the standards GA and catechin. Concerning their comparison with literature studies, only Farhadi et al. [23] investigated the scavenging capacity from grape canes. According to the authors, the inhibition percentage of superoxide generation was in parallel with DPPH values, which is in agreement with present results, as in the DPPH and superoxide assays the Loureiro variety presented the highest ability to scavenge free radicals. Additionally, a direct comparison can be performed with the values reported by Barros et al. [24] which reported the same antioxidant activity assays in grape stems from Portuguese varieties, including TN variety from Quinta da Cavadinha located in Vila Real (Northern Portugal). These authors stated that both red and white stem extracts were strong superoxide radical scavengers with stems of red varieties displaying better results: IC_50_ values ranged from 970 to 1330 µg/mL in red varieties and from 1560 to 2010 µg/mL in white varieties. The reported values were at least 11-times higher than the obtained in the present study for vine-canes demonstrating the efficiency of SWE to obtain extracts more powerful to scavenge O_2_^∙−^.

The ORAC assay, performed only in the best extract, allows the evaluation of Loureiro vine-cane capacity to scavenge ROO^∙^. Loureiro extract (0.0026 ± 0.0002) presented significantly lower potential for peroxyl radical scavenging than the positive controls tested (1.119 ± 0.005 and 7.59 ± 0.07 for GA and catechin, respectively). This is the first time that the effect of vine-cane extracts on peroxyl scavenging capacity has been described, providing valuable information on the biological evaluation of vineyard pruning. Although, the comparison with data available in the literature [24] for a similar matrix (Portuguese grape stems) showed significant differences, with grape stems from red varieties being the most reactive cultivars. Indeed, the comparison of results with the ones found in the literature is not easy because these determinations greatly depend on the genetic load, agro-climatic factors, and extraction procedure [7].

In order to screen the potential use of vine-canes in cosmetic formulations, and taking into consideration the differences between the results previously presented and discussed, it is important to employ other type of methods that allow the prescreening of in vivo effects. Therefore, the cellular effects of subcritical water extracts were also evaluated using keratinocyte and fibroblast cell lines.

### 2.2. Cell Viability Assay

**Figure 1:**
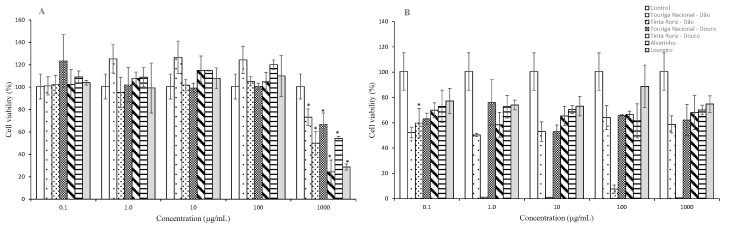
Effect of six vine-canes subcritical water extracts exposure on the viability of HaCaT cells (**A**) and HFF-1 cells (**B**) at different concentrations, as measured by the MTT assay (*n* = 3; * *p* < 0.05 vs. control).

In the present study, vine-cane subcritical water extracts were added to keratinocyte (HaCaT) and fibroblasts (HFF-1) cells to analyze their effects on cell viability (Figure 1). HaCaT and HFF-1 cells are the predominant cell types encountered in the skin, representing the epidermal and dermal layers, respectively, and were selected as in vitro cell models to evaluate the potential toxic effects of producing products for dermatological applications [6,7,22]. These cell types are responsible for skin integrity and, when affected by oxidative stress, are responsible for skin aging [7]. All experiments were carried out up to 1000 μg/mL of extract.

According to the results obtained for the cell viability assays using the HaCaT cell line (Figure 1A), the vine-cane subcritical water extracts did not exhibited cytotoxicity at concentrations up to 100 µg/mL. At the concentration of 1000 µg/mL, it was observed a significant decrease in cell viability of keratinocytes, particularly with TR from the Douro region (24.4%) and Loureiro (28.8%) varieties.

In what concerns to cell viability of HFF-1 (Figure 1B), it was similar for all the tested concentrations of vine-cane extracts, except for TR from Dão region (viability < 7.6% for concentrations above 1 µg/mL.). For extracts concentration of 1000 µg/mL, cell viability values ranged from 58.8% for TN from the Dão region to 74.8% for Loureiro variety.

Through the analysis of the obtained results it was possible to observe that fibroblasts were more sensitive to all vine-cane extracts in comparison to keratinocyte cells, which is in agreement with previous studies [6,22]. The obtained differences between the vine-cane varieties for both cell types could be explained by extracts phenolic composition as they present distinct compound levels with different abilities to permeate or interact with cell membranes. Therefore, according to the obtained results, the optimal range of non-cytotoxic concentrations of each extract to be used in further experiments was up to 1 μg/mL for TR from the Douro region and 100 μg/mL for the other vine-cane varieties tested. Loureiro variety seems to exert the highest protective effect on both cell lines, being considered a good candidate to be used in cosmetic formulations. Nevertheless, further studies are needed to explain this potential protective effect.

As far as we know, this is the first study that evaluates the cell effects of vineyard pruning wastes from different varieties against keratinocytes and fibroblasts cells. Therefore, a comparison with other vine-canes studies is not possible. To date, only two studies [25,26] were performed reporting the effect of vineyard pruning extracts on cells viability. Sáez et al. [25] isolated oligostilbenoids from *V. vinifera* L. Pinot Noir grape canes and study it’s in vitro antiproliferative activity against four different cancer cell lines (MCR-5, AGS, SK-MES-1, and J82) by means of the MTT reduction assay. The authors reported that the whole extract has antiproliferative effect in three human cancer cell lines, with the strongest effect against J82. In another study, Jesus et al. [26] also reported the antiproliferative activity of Loureiro vine-cane extract obtained by ohmic heating on different tumor cell lines (HepG2, MDA-MB-231, MCF-7), demonstrating that vineyard pruning wastes are a source of compounds with relevant anticancer activity. Regarding the evaluation against the same cell lines, only Matos et al. [7] reported that microwave pretreated red wine lees extracts resulted in protection percentages of ≥ 32% at an extract concentration of 250 µg/mL.

### 2.3. Gel Formulation Stability Evaluation

The evaluation of the main characteristics of the produced gel includes diverse physical (such as pH, color, texture and rheological behavior) and microbiological properties. Table 3 summarizes the obtained results for the topical formulation prepared with vine-cane subcritical water extract from the Loureiro variety.

#### 2.3.1. Color

The evaluation of color parameter enables to obtain important information about consumers acceptance of the prepared gel formulation. The color values are presented in Table 3. Taking into consideration that L* value can range from 0 to 100 (maximum), the prepared cosmetic presented lower lightness. Concerning the a* and b* parameters, both values were positive which demonstrates a higher tendency for the red and yellow color. The obtained values were expected, as the prepared gel formulation presented a dark color detected by the eye.

#### 2.3.2. pH

The pH value obtained for gel formulation was 4.79 ± 0.01, which was close to skin pH (range from 5.0 to 6.0) [27], ensuring its compatibility for employing in cosmetic products.

#### 2.3.3. Texture

Like the color parameter, the measurement of gel formulation texture is an important aspect to take into consideration as it is related to consumers acceptance. The firmness and adhesiveness values of the gel formulation are summarized in Table 3. Pinto et al. [21] also prepared gel formulations with hydro-alcoholic extracts of *Castanea sativa bur* and the obtained values for firmness (ranging from 0.288 ± 0.008 to 0.554 ± 0.013 N) and adhesiveness (ranging from −1.480 ± 0.237 to −0.707 ± 0.0802 N.mm) were different from the ones from our present study. Despite from the mentioned differences, a low firmness and a good adherence was observed for the prepared gel formulation with Loureiro subcritical water extract.

#### 2.3.4. Rheological Behavior

**Figure 2:**
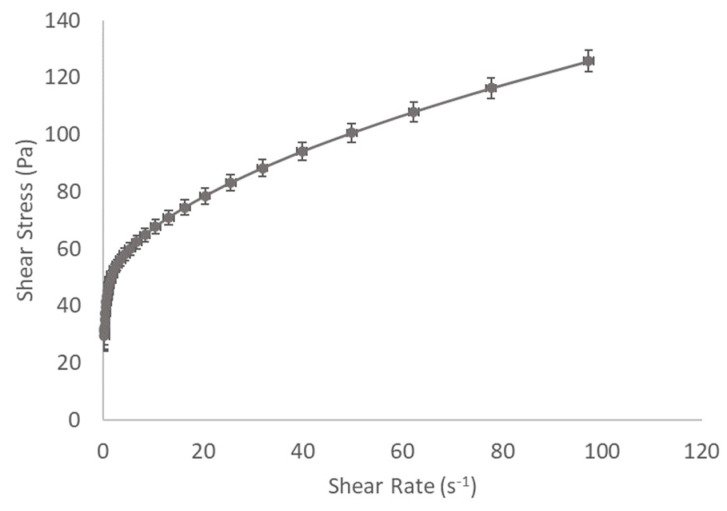
Rheograms of gel formulation prepared from Loureiro subcritical water extract evaluated initially at 25 °C; values were an average of three individual experiments (*n* = 3) expressed as mean ± standard deviation.

Figure 2 represents the shear stress *vs* shear rate for the prepared gel formulation from Loureiro subcritical water extract.

Viscosity is a very important parameter in cosmetic formulations because it can evaluate indirectly the perception felt by users when they apply the product and spread it on the skin. It also gives information about the capacity of release of the active compounds from the final products. As it can be seen from results showed in Figure 2, the gel formulation produced presented a shear thinning behavior, with yield stress, since apparent viscosity decreased with increasing shear rate. The yield stress is the minimal stress value for the gel to flow and this is usually an advantage because without the application of “stress” (at rest) the product does not drain from the skin staying at the site of application. This rheological behavior is characteristic of gels prepared with acrylic polymers as well as water in oil emulsions, being positively evaluate by final consumers since it spreads through the skin as it is rubbed. Indeed, a high viscosity of the semisolid formulation could have a positive influence on the stability of extract once it decreases the diffusion rate of oxygen, as previously reported by our team [21].

#### 2.3.5. Microbiological Properties

The results obtained for microbiological properties at initial time are summarized in Table 3. The results are according to cosmetic legislation since the values obtained for the three groups of microorganisms evaluated were lower than 10 CFU/g gel, revealing no signs of microbial contamination, indicating that a proper preservative system was used in gel formulation.

## 3. Materials and Methods

### 3.1. Chemicals and Reagents

Sodium carbonate (≥99%), Folin’s phenol reagent, gallic acid (GA, ≥98%) and (+)-catechin hydrate (≥98%) were obtained from Sigma-Aldrich (Madrid, Spain). DPPH radical, TPTZ (2,4,6-Tris(2-pyridyl)-s-triazine, 99%), aluminium chloride hexahydrate (99%) and 6-hydroxy-2,5,7,8-tetramethylchromane-2-carboxylic acid (Trolox, 98%) were also from Sigma. Absolute anhydrous ethanol (p.a.) was acquired from Carlo Erba (Peypin, France). Sodium nitrite (≥97%) and potassium dihydrogen phosphate (≥99.5%) were obtained from Merck (Darmstadt, Germany). 3-(4,5-dimethylthiazol-2-yl)-5-(3-carboxymethoxyphenyl)-2-(4-sulfophenyl)-2*H*-tetrazolium (MTT) was obtained from Atom Scientific (Manchester, UK). Iron(III)chloride-6-hydrate (≥99%) and ascorbic acid (AA, 99.7%) were obtained from Riedel-de Haën (Seelze, Germany). Phenazine methosulphate (PMS, ≥ 98%), β-nicotinamide adenine dinucleotide (NADH, ≥95%), nitroblue tetrazolium chloride (NBT, 98%), α,α’-Azodiisobutyramidine dihydrochloride (AAPH, 97%) and fluorescein sodium salt were obtained from Sigma Aldrich (Steinheim, Germany).

Glycerine and (−)-epicatechin were obtained from Fluka (Steinheim, Germany). Carbopol® 940 and triethanolamine were purchased from Vaz Pereira (Lisbon, Portugal). Methanol and formic acid for HPLC analysis were gradient grade and obtained from Merck. Phenolic compound standards were bought to Sigma-Aldrich (Spain) and their purity was at least above 95%. Other chemicals were from Sigma (Spain) and analytical grade.

Dulbecco’s modified Eagle’s médium (DMEM) with GlutaMAX^-I^, fetal bovine serum (FBS), streptomycin, penicillin and amphotericin B were from Invitrogen (Carlsbad, CA, USA). The 3-(4,5-Dimethylthiazol-2-yl)-5-(3-carboxymethoxyphenyl)- 2–4-sulfophenyl)-2*H*-tetrazolium (MTS) assay kit (CellTiter 96 Aqueous One Solution Cell Proliferation Assay) was purchased from Promega (Madison, WI, USA).

### 3.2. Vine-Cane Samples

Vine-canes from six different *Vitis vinifera* varieties (Table 4) used in this work were kindly supplied by Sogrape Vinhos, S. A. (Porto, Portugal) and collected in Quinta dos Carvalhais (Dão region), Quinta do Seixo (Douro region) and Quinta de Azevedo (Minho region) in February 2017 by randomized selection. The moisture content of each vine-cane variety was determined using a Moisture Analyser (MLS 50-3IR160, Kern, Balingen-Fromemmern, Germany) and values are reported in Table 4. After drying the vine-canes at 50 °C for 24 h, they were milled (ZM200, Retsch, Porto, Portugal) to a particle size smaller than 1 mm and stored in sealed bags at room temperature until use. Results were expressed as dry weight (dw) of material.

### 3.3. Subcritical-Water Extraction

SWE was performed in a house-made batch-type extractor as described by Moreira et al. [3]. Extractions were carried out using a solid:liquid ratio of 1:40 *w*/*v* at 150 °C for 40 min under a constant nitrogen pressure of 40 bars. A vibrational platform (frequency of 3 Hz) was used to ensure the agitation of the sample. Extracts were filtered and stored at −20 °C for future analysis. From each vine-cane extract, samples were lyophilized and used for ROS scavenging and cell viability assays. All extractions were carried out in triplicate.

### 3.4. Phytochemical Characterization

#### 3.4.1. Total Phenolic and Flavonoid Content

Vine-cane subcritical water extracts were evaluated for the TPC according to the Folin-Ciocalteau method as described by Moreira et al. [3] using GA as standard (linearity range: 10–150 mg/L, R^2^ > 0.9998). The results were expressed as mg of GA equivalents (GAE) per g of dw of sample (mg GAE/g dw). The TFC was determined spectrophotometrically according to the aluminum chloride procedure previously validated [3]. Epicatechin was used as reference to plot the standard curve (linearity range: 5–150 mg/L, R^2^ > 0.9992) and the results were expressed as mg of epicatechin equivalents per g dw of sample (mg EE/g dw). Each extract was analyzed in triplicate.

#### 3.4.2. HPLC-DAD Analysis of Individual Phenolic Compounds

The six vine-cane varieties were analyzed by HPLC for the phenolic profile characterization. For this analysis, an HPLC system (Shimadzu Corporation, Kyoto, Japan) with a 250 mm × 4.6 mm Gemini C_18_ column from Phenomenex (Alcobendas, Spain) was used according to the method described by Moreira et al. [28]. The elution was carried out with a gradient of methanol (A) and water (B) with 0.1% formic acid at a flow rate of 1.0 mL/min. The detection was recorded with a photodiode array detector at 280, 320 and 360 nm. An aliquot of vine-cane extracts was concentrated 10 times, filtered and 20 µL was injected onto the column. The quantification of polyphenols was made via external calibration (1–200 mg/L) of each standard prepared in a mixture of methanol:water (50/50, *v*/*v*) from the respective stock solution. Each extract was analyzed three times, and the results were expressed as mg of compound/100 g of dw.

### 3.5. Assessment of the Antioxidant Activity

#### 3.5.1. FRAP and DPPH Assays

The antioxidant activity of the vine-cane extracts was evaluated by means of two complementary in vitro methods, namely FRAP and DPPH assays, as described in detail by Moreira et al. [3]. In FRAP assay, AA solutions were used for calibration (linearity range: 10–100 mg/L, R^2^ > 0.9997) and the results were expressed as mg AA equivalents per g of dw (mg AAE/g dw) of sample. In DPPH-RSA assay, a calibration curve was prepared with Trolox (linearity range: 10–150 mg/L, R^2^ > 0.9995) and the antioxidant activity was expressed as mg Trolox equivalents per g of dw (mg TE/g dw) of sample. Each extract was analyzed in triplicate.

#### 3.5.2. Reactive Oxygen Species Scavenging Assays

The antioxidant activity of the extracts was also assessed towards different ROS, namely superoxide and peroxyl radicals. Vine-cane extracts and the GA and catechin standards were dissolved in phosphate buffer. The IC_50_ values (the concentration required to obtain an inhibition capacity of 50%) were obtained from the curves of percentage of inhibition *vs* extract concentration, using the GraphPad Prism 7 software (GraphPad Inc., La Jolla, CA, USA).

#### 3.5.3. Superoxide Radical Scavenging Assay

Scavenging capacities of vine-canes extracts from the six varieties and positive controls (GA and catechin) against the superoxide radical (O_2_^∙−^) were determined using a colorimetric method, based on the reduction of NBT to diformazan, as described by Almeida et al. [20]. These ROS were generated through the non-enzymatic NADH/PMS/O_2_ system, and the absorbance was read at 560 nm for 5 min at 37 °C. Results were expressed as the inhibition, in IC_50_, of the NBT reduction to diformazan.

#### 3.5.4. Peroxyl Radical Scavenging Assay

The peroxyl radical scavenging activity, also known as the oxygen radical absorbance capacity (ORAC) assay, measures the scavenging capacity of ROO^∙^ by monitoring the effect of the selected vine-cane extract on the fluorescence decay resulting from ROO^∙^-induced oxidation of fluorescein [20]. Only the vine-cane extract that presented the highest antioxidant activity as well as the best cell viability was used in this assay. ROO^∙^ was generated by thermo decomposition of AAPH. After preparing the reaction mixture, the 96-well plate was incubated in the microplate reader during 2 h at 37 °C measuring the fluorescence decay every minute. The excitation and emission wavelengths used were 528 ± 20 nm and 485 ± 20 nm, respectively. GA and catechin were used as positive controls. Trolox was used as standard control and the results were expressed in ratio values: slope of the sample/slope obtained for Trolox. All the analyses were performed in triplicate.

### 3.6. Cell Viability Assay

To ensure the quality of vine-cane extracts it is very important to evaluate their cytotoxic potential. To the best of our knowledge, any study has been done regarding subcritical water extracts of different vine-canes varieties. Triplicate wells were incubated with fresh medium in the absence or presence of extracts dissolved in cell culture medium containing 0.1, 1, 10, 100 and 1000 µg/mL of extracts. Extracts dissolved in culture medium were filtered on 0.45 µm Millex GV filters (Millipore, Nepean, ON, Canada) and exposed to keratinocytes (HaCaT) and fibroblasts (HFF-1) cells during 24 h.

#### 3.6.1. Cell Lines, Primary Cell Isolation and Culture Conditions

HaCaT and HFF-1 cells were obtained from American Type Culture Collection (ATCC, Barcelona, Spain). Passage 28–30 of HaCaT and passage 53–55 of HFF-1 were used for the MTT assay, which enables to monitor the uptake and metabolism of the vital mitochondrial dye by cell mitochondria. Cells were grown in Dulbecco’s modified Eagle’s medium (DMEM) with GlutaMAXTM-I (Invitrogen, Thermo Fisher Scientific, California, USA) supplemented with 10% fetal bovine serum (FBS), 100 U/mL penicillin and 100 μg/mL streptomycin, and 0.25 μg/mL amphotericin B, and maintained in a 5% CO_2_ environment at 37 °C. At 90–95% confluence, both cell lines were harvested using trypsin (Invitrogen) and re-plated in T125 flasks.

#### 3.6.2. MTT Assay

The MTT assay was performed according to the manufacturer instructions. Briefly, cells were cultured in 96-well microtiter plate at a density of 25 × 10^3^ cells per mL culture medium for 24 h. Then, the cells were incubated with 0.1–1.0 mg/mL of vine-cane extracts for 24 h at 37 °C. The positive control was DMEM and the negative control was 1% (*w*/*v*) Triton X-100. Following the removal of extracts from the wells, cells were washed with HBSS. The number of viable cells was then evaluated by adding the MTT reagent in medium to each well and incubating for 3 h at 37 °C. DMSO was used to solubilize the crystals. Control was determined incubating cells with culture medium. The absorbance was measured at 590 nm with background subtraction at 630 nm. Each concentration was tested in triplicate in three independent experiments. Results were expressed as percentages of cell viability.

### 3.7. Cosmetic Formulation

The topical formulation selected to incorporate the subcritical water extract from Loureiro vine-cane variety was a gel, and was produced as previously described by Pinto et al. [21] with slight modifications. The gel was formulated with the following components: glycerin (7%), carbopol (0.5%), triethanolamine (0.3%), preservative (phenoxyethanol/methyl paraben/ethyl paraben/ propyl paraben/butyl paraben mixture, 0.1%), and perfume (0.1%). The gel was prepared by dissolving carbopol in the extract (75%)/water (17%) mixture at room temperature. After the addition of other ingredients, gelation process was initiated by the addition of triethanolamine by continuous stirring to form a homogenous gel. The gels were prepared in triplicate, packed in polyethylene jars, and rested for 24 h before measurements.

### 3.8. Formulation Stability Evaluation

The physical and microbiological properties of the prepared formulation were evaluated at the initial time using the procedures described below.

#### 3.8.1. Color

Color was evaluated with a colorimeter (CR-400, Konica Minolta, Tokyo, Japan) and results were expressed according to the color space CIE 1976 L*a*b*, defined by the Comission Internationale de l’E’clairage (CIE). The three coordinates represent the lightness of the color (L*), its position between red and green (a*) and its position between yellow and blue (b*). The color space values were registered using the Spectra Magic Nx data software (version CM-S100W 2.03.0006, Konica Minolta). Measurements were performed in triplicate.

#### 3.8.2. pH

The pH was measured by a pH meter (micropH 2002 meter, Crison, Alella, Barcelona) equipped with a glass electrode after mixing 1 g of gel formulation with 3 mL of water. The measurements were performed in triplicate.

#### 3.8.3. Texture Analysis

Texture analysis was carried out in a texturometer (TA-XT2i Texture Analyser from Stable Micro Systems, Vienna Court, UK). A penetration test was performed at 25 °C using the Radiused Cylinder Probe P/0.5 probe (Stable Micro Systems). The applied force of the probe was calculated at the penetration depth of 5 mm with a test speed of 1 mm/s (trigger value of 0.005 N). All measurements were done in triplicate, in different parts of the gel formulation. From the graph force *vs* distance, the maximum force (N) corresponds to the firmness and the negative area (N·mm) corresponds to the adhesiveness.

#### 3.8.4. Rheological Measurements

Rheological properties of the formulation were measured at 25 °C using the Kinexus lab + Rheometer from Malvern Panalytical (Malvern, UK), equipped with the Peltier Plate Cartridge and the CP4/40 SR4321 SS: PL61 S2874 SS concentric cylinder geometry. To measure the viscosity of the gel, the start and end shear rates applied were 0.1 and 100 s^−1^ respectively, with a ramp time of 5 min, collecting 10 samples per decade. Viscosity measurements were performed in triplicate and results were expressed as mean ± standard deviation (SD). Thixotropic properties of the gel were evaluated based on a rotational 3-step thixotropy test. In the first phase a shear rate of 0.1 s^−1^ for 2 min was applied to the gel, collecting data every 5 s. In the second phase, a much higher shear rate, 100 s^−1^, was applied for 30 s collecting data every 5 s. At the end, gel structure was allowed to recover by applying again a shear rate of 0.1 s^−1^ for 20 min and collecting data every 5 s. Yield stress values were obtained at the viscosity peak by applying a shear stress ranging from 0.0 to 200.0 Pa for 5 min. Data was collected using the rSpace for Kinexus software (version 1.75.2326, Malvern Instruments.).

#### 3.8.5. Microbiological Analysis

Microbiological quality of the formulation was evaluated regarding the total mesophilic aerobic bacteria, yeast and mould content at initial time. Dilutions were performed with buffered peptone sterile solution and 3% *w*/*v* of polysorbate 80 was added to the culture media to neutralize the preservative. Total mesophilic aerobic bacteria count was performed using nutrient agar medium, after 5 days of incubation at 32.5 °C. For yeast and moulds counting, Sabouraud dextrose agar was used and incubation was carried out during 5 days at 22.5 °C. Results were expressed as number of colony-forming units per g (CFU/g).

### 3.9. Statistical Analysis

IBM SPSS Statistics for Windows software (Version 24.0, IBM Corp., Armonk, NY, USA) was used for the statistical analysis. Data was reported as mean ± SD of three replications. The normal distribution and the homogeneity of variances were assessed by Shapiro-Wilk’s and Levene’s tests, respectively. For all assays, the data were normal and the homogeneity of variances confirmed. To evaluate the differences between samples, the one-way ANOVA was used. Tukey’s HSD test was employed for the *post hoc* comparisons of the means, being *p* < 0.05 accepted as denoting significance. To compare the same sample at different temperatures, a *t*-test was employed, being *p* < 0.05 accepted as denoting significance. GraphPad Prism 7 software was employed to calculate the IC_50_ values of ROS scavenging activity.

## 4. Conclusions

The results of this work underline that vine-canes from different grapes varieties represent a good source of natural polyphenolic compounds. Loureiro subcritical water extract revealed better results than all the other tested extracts in terms of scavenging capacity against different reactive species as well as regarding the effects on human skin cells viability. Additionally, a gel formulation with 75% of Loureiro subcritical water extract was successfully produced. Physical and microbiological properties analysis demonstrated that the produced cosmetic is suitable for skin application and, consequently, enhances the consumer acceptance. As a future perspective, this formulation could be used to increase the protection against different oxidants. In this way, further investigations are being performed to explore the in vivo effects of the gel containing Loureiro subcritical water extract. However, these results open ups a new market possibility for the re-use of vineyard pruning wastes as cosmetic products with antioxidant and anti-aging effects.

## Figures and Tables

**Table 1:** Phytochemical composition and antioxidant activity of vine-cane subcritical water extracts from Touriga Nacional (TN) and Tinta Roriz (TR) varieties from Dão and Douro regions, Alvarinho and Loureiro varieties; results are expressed as mean ± standard deviation (*n* = 6).

	Phytochemical Composition		Antioxidant Activity		
	TPC (mg GAE/g dw)	TFC (mg EE/g dw)	FRAP (mg AAE/g dw)	DPPH (mg TE/g dw)	O_2_^∙−^ IC_50_ (µg/mL)
**Vine-cane variety**					
TN Dão	30.82 ± 3.04 ^a, b^	8.25 ± 0.65 ^b^	29.99 ± 1.83^c^	5.95 ± 0.44 ^c^	84.78 ± 4.48 ^c^
TR Dão	31.33 ± 2.11 ^a, b^	8.07 ± 0.79 ^b^	38.33 ± 2.0 ^a, b^	6.40 ± 0.53 ^c^	59.96 ± 2.79 ^a, b^
TN Douro	32.49 ± 2.70 ^a^	9.77 ± 0.67 ^b, c^	35.72 ± 2.10^b^	10.23 ± 0.70 ^b^	75.38 ± 9.09 ^a, b, c^
TR Douro	32.42 ± 1.87 ^a^	10.07 ± 0.94 ^a^	40.65 ± 2.18 ^a^	10.99 ± 0.51 ^b^	79.99 ± 4.94 ^b, c^
Alvarinho	27.38 ± 1.40 ^c^	6.03 ± 0.34 ^d^	35.83 ± 1.90 ^b^	17.21 ± 0.65 ^a^	61.52 ± 5.64 ^a, b^
Loureiro	29.26 ± 1.47 ^b, c^	7.00 ± 0.67 ^c, d^	36.81 ± 1.65 ^b^	17.89 ± 093 ^a^	56.67 ± 2.59 ^a^
**Positive controls**					
Gallic acid					8.70 ± 0.70 ^e^
Catechin					23.98 ± 1.39 ^d^

TPC, total phenolic content; TFC, total flavonoid content; FRAP, ferric reducing antioxidant power; DPPH, 1,1-diphenyl-2-picrylhydrazyl assay; O_2_^∙−^, superoxide anion radical scavenging assay; dw: dry weight; GAE, gallic acid equivalents; EE, epicatechin equivalents; AAE, ascorbic acid equivalents; TE, Trolox equivalents; IC_50_, in vitro inhibitory concentration, expressed in μg/mL, required to scavenge 50% of the generated reactive oxygen species (mean ± SD, *n* = 3). Different letters in the same column indicate significant differences (*p* < 0.05) between samples.

**Table 2:** Individual phenolic compounds identified in vine-cane subcritical water extracts; results are expressed as means ± standard deviation (mg of compound per 100 g dw, *n* = 3).

Vine-Cane Variety	TN Dão	TR Dão	TN Douro	TR Douro	Alvarinho	Loureiro
Gallic acid	527 ± 26	895 ± 45	546 ± 27	1014 ± 51	568 ± 28	802 ± 40
Protocatechuic acid	41.7 ± 2.1	24.9 ± 1.2	28.0 ± 1.4	33.0 ± 1.6	23.8 ± 1.2	18.5 ± 0.9
(+)-Catechin	225 ± 11	132 ± 7	156 ± 8	144 ± 7	115 ± 6	289 ± 14
4-hydroxyphenilacetic acid	19.9 ± 1.0	7.63 ± 0.38	22.1 ± 1.1	7.85 ± 0.39	19.2 ± 1.0	6.76 ± 0.34
4-hydroxybenzoic acid	3.70 ± 0.18	1.78 ± 0.09	4.58 ± 0.23	2.15 ± 0.11	4.28 ± 0.21	1.71 ± 0.09
4-hydroxybenzaldehyde	16.3 ± 0.8	10.4 ± 0.5	16.7 ± 0.8	10.3 ± 0.5	17.2 ± 0.9	9.42 ± 0.54
Chlorogenic acid	23.8 ± 1.2	14.5 ± 0.7	25.6 ± 1.3	25.0 ± 1.2	25.1 ± 1.3	24.7 ± 1.2
Vanillic acid	30.6 ± 1.5	1.64 ± 0.08	30.1 ± 1.5	14.5 ± 0.7	29.1 ± 1.5	1.68 ± 0.08
Caffeic acid	11.1 ± 0.6	7.33 ± 0.37	13.1 ± 0.7	13.1 ± 0.7	14.2 ± 0.7	13.9 ± 0.7
Syringic acid	42.9 ± 2.1	38.6 ± 1.9	39.0 ± 1.9	36.6 ± 1.8	38.9 ± 1.9	31.0 ± 1.6
(−)-Epicatechin	31.5 ± 1.6	20.4 ± 1.0	30.1 ± 1.5	21.6 ± 1.1	32.6 ± 1.6	21.2 ± 1.1
*p*-Coumaric acid	3.77 ± 0.19	3.87 ± 0.19	3.72 ± 0.19	3.71 ± 0.19	3.66 ± 0.18	3.89 ± 0.19
Ferulic acid	17.0 ± 0.8	10.4 ± 0.5	17.7 ± 0.9	12.7 ± 0.6	15.3 ± 0.8	10.6 ± 0.5
Sinapic acid	10.2 ± 0.5	36.2 ± 1.8	13.9 ± 0.7	11.5 ± 0.6	10.2 ± 0.5	19.4 ± 1.0
Naringin	19.1 ± 1.0	35.1 ± 1.8	30.0 ± 1.5	26.9 ± 1.3	31.6 ± 1.6	27.8 ± 1.4
Ellagic acid	11.4 ± 0.6	25.1 ± 1.3	33.5 ± 1.7	31.0 ± 1.6	24.8 ± 1.2	32.4 ± 1.6
Rutin	9.45 ± 0.47	7.59 ± 0.38	19.6 ± 0.9	26.6 ± 1.3	11.5 ± 0.6	27.2 ± 1.4
Resveratrol	8.92 ± 0.45	11.7 ± 0.6	18.6 ± 0.9	19.5 ± 0.9	16.6 ± 0.8	17.3 ± 0.9
Quercetin-3-*O*-glucopyranoside	11.7 ± 0.6	28.3 ± 1.4	8.58 ± 0.43	14.4 ± 0.7	11.9 ± 0.6	12.8 ± 0.6
Phloridzin	2.67 ± 0.13	9.10±0.46	2.75 ± 0.14	3.44 ± 0.17	2.53 ± 0.13	2.92 ± 0.15
Cinnamic acid	4.75 ± 0.24	6.41 ± 0.32	4.75 ± 0.24	4.84 ± 0.24	4.68 ± 0.23	4.78 ± 0.24
Myricetin	59.4 ± 3.0	61.0 ± 3.1	58.3 ± 2.9	59.2 ± 3.0	56.2 ± 2.8	60.0 ± 3.0
Kaempferol-3-*O*-glucoside	8.60 ± 0.43	12.4 ± 0.6	8.15 ± 0.41	8.73 ± 0.44	7.95 ± 0.40	8.53 ± 0.43
Kaempferol-3-*O*-rutinoside	10.1 ± 0.5	14.0 ± 0.7	8.78 ± 0.44	8.51 ± 0.43	8.48 ± 0.42	8.64 ± 0.43
Naringenin	2.17 ± 0.11	2.53 ± 0.13	1.93 ± 0.10	2.37 ± 0.12	2.00 ± 0.10	2.35 ± 0.12
Quercetin	16.1 ± 0.8	16.0 ± 0.8	16.2 ± 0.8	16.1 ± 0.8	15.7 ± 0.8	21.0 ± 1.1
Phloretin	2.04 ± 0.10	2.22 ± 0.11	2.01 ± 0.10	2.14 ± 0.11	ND	2.02 ± 0.10
Tiliroside	7.79 ± 0.39	7.88 ± 0.39	7.46 ± 0.37	6.93 ± 0.35	7.34 ± 0.37	7.18 ± 0.36
Kaempferol	22.9 ± 1.1	19.4 ± 0.9	19.5 ± 0.9	21.4 ± 1.1	18.1 ± 0.9	22.7 ± 1.1
Pinocembrin	8.50 ± 0.43	8.23 ± 0.41	8.23 ± 0.41	8.29 ± 0.41	ND	8.24 ± 0.41
Σ Phenolic acids	764 ± 38	1084 ± 54	799 ± 40	1220 ± 61	798 ± 40	981 ± 49
Σ Flavonoids	432 ± 22	365 ± 18	373 ± 19	365 ± 18	318 ± 16	516 ± 26
Σ Stilbenes	8.92 ± 0.45	11.7 ± 0.6	18.6 ± 0.9	19.5 ± 1.0	16.6 ± 0.8	17.3 ± 0.9
Σ Others	4.71 ± 0.24	11.3 ± 0.6	4.76 ± 0.24	5.59 ± 0.28	2.53 ± 0.13	4.94 ± 0.25
Σ All phenolic compounds	1210	1472	1195	1610	1135	1520

ND, not detected.

**Table 3:** Physical and microbiological properties of gel formulation prepared with Loureiro subcritical water extract evaluated at 25 °C at initial time; values are means ± SD (*n* = 3).

**Color**	L*	25.38 ± 1.19
	A*	6.53 ± 0.30
	B*	16.55 ± 0.97
**pH**		4.79 ± 0.01
**Texture**	Firmness (N)	0.060 ± 0.001
	Adhesiveness (N·mm)	−0.102 ± 0.007
**Microbiological properties**	Aerobic Bacteria Count (CFU/g gel)	<1.0 × 10^1^
	Total Yeast Count (CFU/g gel)	<1.0 × 10^1^
	Total Mould Count (CFU/g gel)	<1.0 × 10^1^

L*, lightness; A*, red to green; B*, yellow to blue. CFU, colony forming units.

**Table 4:** Portuguese vine-cane varieties characteristics.

Sample Code	Vine-Cane Variety	Origin	Moisture Content (%)
TN Dão	Touriga Nacional	Dão region	14.0 ± 0.5
TR Dão	Tinta Roriz	Dão region	11.6 ± 0.1
TN Douro	Touriga Nacional	Douro region	10.7 ± 0.6
TR Douro	Tinta Roriz	Douro region	7.3 ± 0.6
Alvarinho	Alvarinho	Minho region	11.0 ± 0.6
Loureiro	Loureiro	Minho region	9.5 ± 0.6
